# Real-world effectiveness of pangenotypic direct-acting antivirals in chronic hepatitis C: A retrospective cohort study

**DOI:** 10.1097/MD.0000000000050910

**Published:** 2026-09-25

**Authors:** Berfin Çirkin Doruk, Mustafa Uğuz

**Affiliations:** a Department of Infectious Diseases and Clinical Microbiology, Mersin City Training and Research Hospital, Mersin, Türkiye.

**Keywords:** direct-acting antivirals, hepatitis C, pangenotypic therapy, real-world data, sustained virologic response

## Abstract

The aim of this study was to evaluate virological response, biochemical changes, and treatment tolerability in patients with chronic hepatitis C receiving pangenotypic direct-acting antiviral (DAA) therapy. The study included patients with chronic hepatitis C virus infection followed at a tertiary care center between 2018 and 2023. Data were evaluated retrospectively. Patients who completed pangenotypic DAA treatment and had an available SVR12 (sustained virologic response 12 weeks after treatment completion) result were included in the study. Demographic characteristics, genotype distribution, presence of cirrhosis, previous treatment history, hepatitis C virus RNA results, biochemical parameters, and safety data were analyzed descriptively. Fifty-seven patients were included in the study. The mean age was 44.1 years. Thirty-seven patients were male, and 3 patients had compensated cirrhosis. The most frequently detected genotype was 1b, followed by genotype 3a. SVR12 was obtained in all evaluable patients. No virological breakthrough, relapse, treatment discontinuation, or serious adverse events were observed. During treatment, alanine aminotransferase and total bilirubin levels decreased significantly. Albumin levels, however, increased. In this single-center real-world cohort, pangenotypic DAA treatment was associated with SVR12 in all evaluable patients. Treatment was well tolerated, and early biochemical improvement was achieved. The findings should be interpreted considering the retrospective design of the study and the small subgroup size.

**Key points**

- Pangenotypic DAA treatment achieved SVR12 in all evaluable patients.
- ALT and total bilirubin levels decreased, whilst albumin levels increased.
- No virological breakthroughs, relapses, or serious adverse events were observed.
- These results indicate that pangenotypic DAA regimens are effective and well tolerated in routine clinical practice.

## 1. Introduction

Chronic hepatitis C virus (HCV) infection remains a major global health challenge. The World Health Organization estimates that over 58 million people are chronically infected, with around 1.5 million new cases occurring each year. A considerable proportion of these cases remain undiagnosed or untreated.^[1]^ Without effective antiviral therapy, the infection progresses to fibrosis, cirrhosis, hepatic decompensation, and hepatocellular carcinoma, resulting in significant liver-related morbidity and mortality.^[2,3]^ In response to this burden, the World Health Organization has endorsed a global elimination strategy emphasizing timely diagnosis, broad treatment access, and sustained virologic cure by 2030.^[1]^

The advent of direct-acting antiviral (DAA) drugs that do not contain interferon has radically transformed the treatment of chronic HCV. Pangenotypic treatment regimens have demonstrated a short treatment duration, excellent tolerability, and cure rates exceeding 95% in clinical trials conducted across various patient groups.^[4–6]^ Consequently, current international guidelines recommend pangenotypic DAAs as first-line treatment, including for patients with compensated cirrhosis or those who have previously undergone treatment.^[2,3]^

Although the guidelines are based on strong evidence, real-world outcomes may differ from those observed in controlled trials. Treatment adherence, comorbidities, the presence of cirrhosis, and access to healthcare may influence this difference.^[6–9]^ Observational studies have reported resolution of liver inflammation and improvement in biochemical test results following viral eradication; however, the extent of this improvement may vary between patient groups.^[6–9]^ Therefore, real-world data support the implementation of guidelines into daily practice, particularly in regions where access to DAA treatments has increased.

The seroprevalence of HCV in Türkiye is approximately 0.5% to 1.0%, with genotype 1b being the predominant genotype.^[10–12]^ Access to oral DAA treatments has increased since 2018 following changes to reimbursement regulations. However, real-world data reported from Türkiye are generally based on limited patient cohorts or a single treatment regimen.^[10–12]^ Consequently, there remains a need for studies that evaluate treatment success, biochemical response, and subgroup outcomes together in routine clinical practice.

We therefore evaluated HCV patients who were followed up at a tertiary center and who had completed their 12-week posttreatment follow-up. Our aim was to investigate the real-world efficacy and safety of 2 pangenotypic DAA regimens, demonstrate biochemical changes during treatment, and assess whether the SVR12 (sustained virologic response 12 weeks after treatment completion) outcome varied according to demographic or clinical characteristics. The study provides up-to-date real-world data from Türkiye.

## 2. Materials and methods

### 2.1. Study design and setting

This retrospective observational cohort study was conducted at a tertiary care hospital. Records of patients followed up for chronic HCV infection between January 2018 and December 2023 were reviewed. Data were obtained from electronic patient records and follow-up files held by the infectious diseases clinic. The study protocol was approved by the Mersin University Ethics Committee. Due to the retrospective design and the use of anonymized data, written patient consent was not required.

### 2.2. Population and patient flow

In our study, patients over 18 years of age diagnosed with chronic HCV infection were screened from the database. Chronic HCV infection was defined as HCV RNA positivity detected by real-time polymerase chain reaction. As a result of the screening, 154 patients were identified and formed the study population. The patients were divided into 4 groups:

1. HCV RNA–negative patients without treatment indication;
2. RNA-positive individuals in whom treatment was planned but not initiated;
3. Patients who started treatment but lacked complete follow-up; and
4. Patients who completed DAA therapy and posttreatment evaluation.

The analytic cohort consisted of patients in group 4 (n = 57), for whom the virological endpoint was fully assessable. A patient flow summary describing cohort formation is presented in Table 1.

**Table 1:** Baseline demographic and clinical characteristics of the total patient pool and analytic cohort.

Variable	Total pool (n = 154)	Analytic cohort (n = 57)*
Age (yr, mean ± SD)	45.2 ± 14.8	44.1 ± 13.9
Male sex, n (%)	95 (61.7)	37 (64.9)
Cirrhosis, n (%)	23 (14.9)	3 (5.3)
Previous antiviral therapy, n (%)	11 (7.1)	5 (8.8)
HCV RNA positivity, n (%)	114 (74.0)	57 (100)
Genotype 1b, n (%)	98 (63.6)	34 (59.6)
Genotype 3a, n (%)	33 (21.4)	13 (22.8)
Other genotypes, n (%)	23 (15.0)	10 (17.6)
Sofosbuvir/velpatasvir/voxilaprevir (Vosevi), n (%)	–	41 (71.9)
Glecaprevir/pibrentasvir (Maviret), n (%)	–	16 (28.1)
Completed treatment, n (%)	57 (37.0)	57 (100)
Treatment discontinuation/serious adverse events	0	0
SVR12 rate (%; 95% CI)	–	100 (93.7–100)

The total patient pool included HCV RNA–negative patients without a treatment indication (n = 40), RNA-positive patients in whom treatment was planned but not initiated (n = 10), patients who initiated treatment but lacked complete follow-up data (n = 47), and patients who completed treatment and posttreatment follow-up (n = 57).

CI = confidence interval, HCV = hepatitis C virus, SD = standard deviation, SVR12 = sustained virologic response 12 weeks after treatment completion.

* The analytic cohort included only patients who completed treatment and SVR12 follow-up.

### 2.3. Inclusion and exclusion criteria

Patients were eligible for inclusion in the analytic cohort if they had confirmed chronic HCV infection documented by detectable HCV RNA, were treated with a pangenotypic DAA regimen, completed the full prescribed therapeutic course, and had an available SVR12 assessment. Patients were excluded if they had human immunodeficiency virus or hepatitis B virus coinfection, a history of liver transplantation, substantially incomplete clinical or laboratory data that prevented reliable evaluation of treatment outcomes, discontinued therapy prematurely, or had unavailable SVR12 results.

The 2 pangenotypic regimens used were the fixed-dose triple combination sofosbuvir/velpatasvir/voxilaprevir (Vosevi), typically administered for 12 weeks, and glecaprevir/pibrentasvir (Maviret), typically administered for 8 weeks. During the study period, sofosbuvir/velpatasvir/voxilaprevir was the accessible and reimbursed sofosbuvir-based pangenotypic regimen in Türkiye. Therefore, it was used as initial therapy in eligible patients, irrespective of previous DAA failure, in accordance with the reimbursement conditions and routine clinical practice applicable at that time. Ribavirin was not used in any patient. Regimen selection was based on viral genotype, prior antiviral exposure, disease severity, treatment availability, reimbursement conditions, and physician judgment. No dose modifications or treatment interruptions occurred during the treatment period.

### 2.4. Laboratory and virological assessments

HCV RNA levels were measured at baseline, treatment month 1, end of treatment (EOT), and approximately 12 weeks after treatment completion. HCV RNA was assessed using real-time polymerase chain reaction with a lower limit of quantification of 15 IU/mL. SVR12 was defined as undetectable HCV RNA 12 weeks after treatment completion.

The biochemical assessment examined alanine aminotransferase (ALT), total bilirubin, and albumin levels, platelet count, and international normalized ratio (INR) values. Where there were multiple measurements of the same parameter within the follow-up period, the measurement closest to the reference time point was included in the analysis.

### 2.5. Outcome definitions

SVR12 was the primary endpoint. Secondary endpoints included changes in biochemical parameters from baseline to the EOT, as well as treatment discontinuation and adverse events (AEs) recorded during follow-up.

### 2.6. Subgroup analyses and handling of missing data

Predefined subgroup analyses were performed to assess treatment response according to age (<65 vs ≥65 years), viral genotype (1b vs 3a), compensated cirrhosis status, previous antiviral treatment exposure, and the pangenotypic regimen administered. Given the modest subgroup sample sizes and the absence of virological failures, these comparisons were interpreted descriptively rather than as inferential subgroup effects. To ensure definitive virological endpoints and outcome accuracy, only patients who completed the full treatment course and had an available SVR12 assessment were included in the analytic cohort; therefore, no imputation procedures were applied. The 154 patients represented the screened source population and were reported to transparently describe cohort assembly and patient disposition; patients were included in the primary effectiveness analysis only if they completed treatment and had an evaluable SVR12 endpoint. Laboratory values missing at specific time points were excluded from paired analyses.

### 2.7. Statistical analysis

Continuous variables were reported as mean ± standard deviation or median (interquartile range) according to distribution. Categorical variables were summarized as counts and percentages. Normality was tested using the Shapiro–Wilk test. A paired *t* test or Wilcoxon signed-rank test was used for longitudinal biochemical measures. Categorical comparisons were performed using the χ^2^ test or Fisher’s exact test.

Two-tailed *P* < .05 was considered statistically significant. Analyses were performed using IBM SPSS Statistics version 28.0 (IBM Corp., Armonk) and R version 4.3.2 (R Foundation for Statistical Computing, Vienna, Austria).

### 2.8. Ethical approval

This retrospective study was approved by the Mersin University Ethics Committee (Decision No. 2023/547; date: September 6, 2023). The study was conducted in accordance with the principles of the Declaration of Helsinki. The requirement for written informed consent was waived by the ethics committee because of the retrospective design of the study and the use of anonymized patient data.

## 3. Results

### 3.1. Patient flow and cohort selection

During the study period, 154 patients with confirmed HCV infection were screened (Table 1). Of these, 40 (26.0%) were HCV RNA–negative and did not require antiviral therapy; 10 (6.5%) were treatment-eligible but did not initiate therapy; and 47 (30.5%) initiated treatment but lacked complete follow-up. The final analysis included 57 patients (37.0%). No additional loss to follow-up occurred among the 57 patients included in the analytic cohort.

### 3.2. Baseline characteristics

Baseline demographic and clinical characteristics are summarized in Table 1. The mean age of the patients was 44.1 ± 13.9 years, and 64.9% (n = 37) were male. The prevalence of compensated cirrhosis was 5.3% (n = 3), while the prevalence of a history of prior antiviral treatment was 8.8% (n = 5). The most common genotype was 1b (59.6%); this was followed by genotype 3a (22.8%) and other genotypes (17.6%). Sofosbuvir/velpatasvir/voxilaprevir was administered to 41 patients (71.9%), whereas glecaprevir/pibrentasvir was administered to 16 patients (28.1%). The distribution of treatment regimens according to HCV genotype is shown in Table 2. Among patients with genotype 1b, 26 received sofosbuvir/velpatasvir/voxilaprevir and 8 received glecaprevir/pibrentasvir; among those with genotype 3a, 9 and 4 patients received these regimens, respectively. For genotype 1a, the corresponding numbers were 4 and 2, and for genotype 2, 2 patients received each regimen. All patients completed the planned treatment without any dose adjustments or treatment interruptions.

**Table 2:** Distribution of pangenotypic DAA regimens according to HCV genotype.

HCV genotype	Sofosbuvir/velpatasvir/voxilaprevir (Vosevi), n (%)	Glecaprevir/pibrentasvir (Maviret), n (%)	Total, n
Genotype 1a	4 (66.7)	2 (33.3)	6
Genotype 1b	26 (76.5)	8 (23.5)	34
Genotype 2	2 (50.0)	2 (50.0)	4
Genotype 3a	9 (69.2)	4 (30.8)	13
Total	**41 (71.9**)	**16 (28.1**)	**57**

Percentages are calculated within each genotype category.

DAA = direct-acting antiviral, HCV = hepatitis C virus.

### 3.3. Virological response

The median baseline HCV RNA level was 2.8 × 10^6^ IU/mL. By the first month of treatment, HCV RNA levels were found to be below the limit of quantification (<15 IU/mL) in 51 of the 57 patients (89.5%). At the EOT and 12 weeks after completion of treatment, all patients tested negative for HCV RNA. The SVR12 rate was 100% (95% confidence interval: 93.7%–100%). No virological breakthrough or relapse was observed.

### 3.4. Biochemical response

Changes in biochemical parameters over time are shown in Table 3.

**Table 3:** Changes in laboratory parameters over time (baseline, month 1, end of treatment).

Parameter	Baseline (mean ± SD)	Month 1 (mean ± SD)	End of treatment (mean ± SD)	*P* value
ALT (U/L)	61 ± 48	38 ± 22	26 ± 14	<.001
Albumin (g/dL)	3.7 ± 0.5	3.9 ± 0.4	4.1 ± 0.3	.002
Total bilirubin (mg/dL)	1.15 ± 0.61	0.91 ± 0.44	0.74 ± 0.35	.031
Platelet count (×10^9^/L)	182 ± 58	188 ± 61	193 ± 64	.221
INR	1.11 ± 0.16	1.09 ± 0.15	1.08 ± 0.14	.287

Values are presented as mean ± standard deviation. *P* values were calculated using the paired *t* test or Wilcoxon signed-rank test, as appropriate.

ALT = alanine aminotransferase, INR = international normalized ratio, SD = standard deviation.

ALT levels decreased from 61 ± 48 U/L at baseline to 26 ± 14 U/L at EOT (*P* < .001), with normalization in 93.0% of patients. Total bilirubin declined from 1.15 ± 0.61 to 0.74 ± 0.35 mg/dL (*P* = .031), while albumin increased from 3.7 ± 0.5 to 4.1 ± 0.3 g/dL (*P* = .002). Platelet count and INR did not show a statistically significant change between baseline and EOT (*P* > .05 for both).

Figure 1 illustrates the trajectories of ALT and bilirubin during therapy.

**Figure 1.**
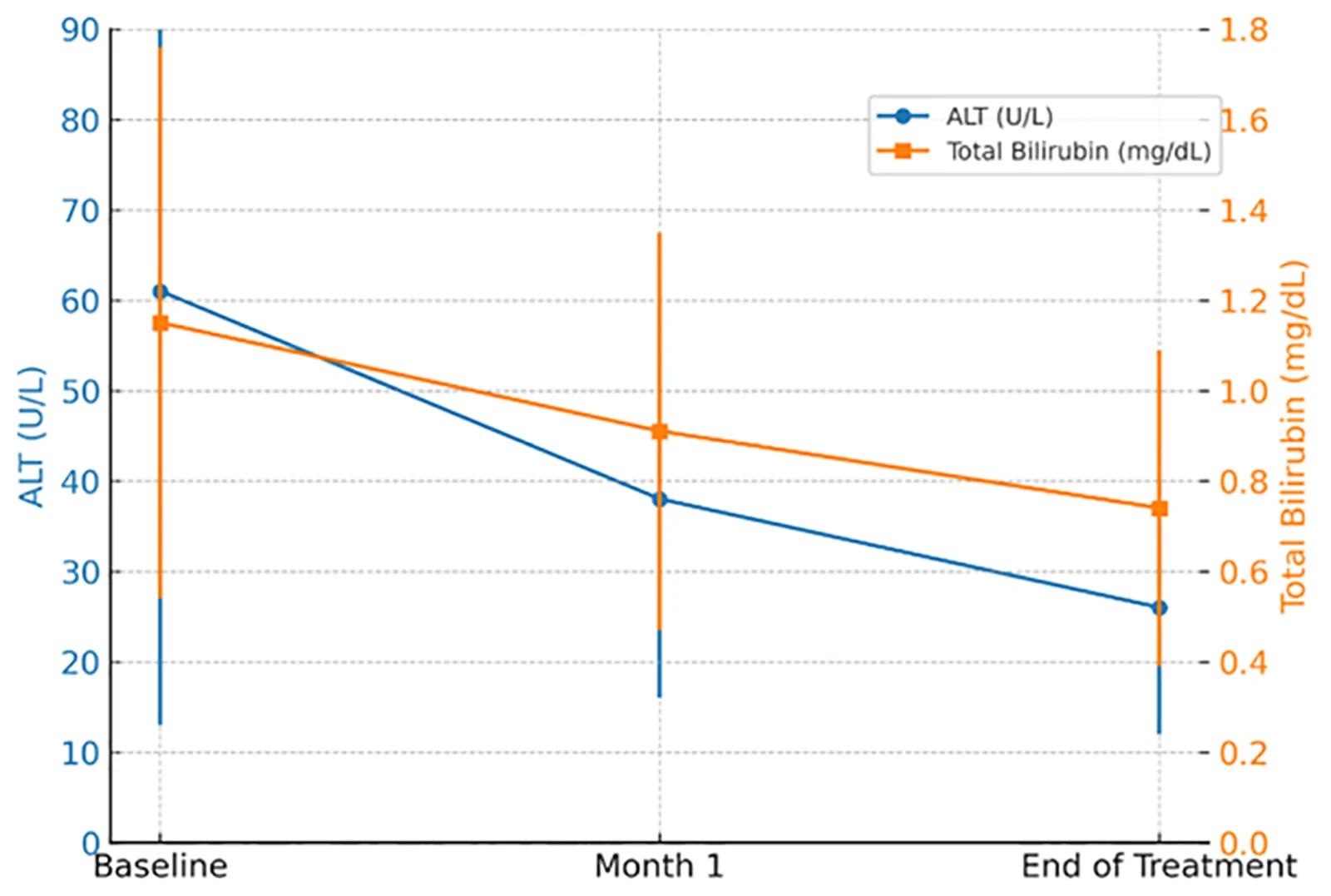
Longitudinal changes in ALT and total bilirubin during direct-acting antiviral therapy. Mean ALT (blue circles, left axis) and total bilirubin (orange squares, right axis) levels measured at baseline, treatment month 1, and EOT. Vertical bars indicate standard deviations. ALT values declined substantially during treatment, and total bilirubin demonstrated a gradual reduction, reflecting biochemical improvement during antiviral therapy. ALT = alanine aminotransferase, EOT = end of treatment.

### 3.5. Subgroup outcomes

Sustained virologic response was achieved in all predefined subgroups, including age ≥65 years, prior treatment exposure, genotypes 1b and 3a, compensated cirrhosis, and both DAA regimens. Because no virological failures occurred and subgroup sizes were limited, these findings were interpreted descriptively (Table 4).

**Table 4:** Sustained virologic response (SVR12) by subgroups.

Subgroup	n	SVR12 (%)
Compensated cirrhosis	3	100
Non-cirrhotic	54	100
Age ≥65 yr	12	100
Age <65 yr	45	100
Treatment-experienced	5	100
Treatment-naïve	52	100
Genotype 1b	34	100
Genotype 3a	13	100
Sofosbuvir/velpatasvir/voxilaprevir	41	100
Glecaprevir/pibrentasvir	16	100

SVR12 was achieved in all listed subgroups. Because no virological failures occurred and subgroup sizes were limited, subgroup findings were interpreted descriptively, and no inferential comparison was performed.

SVR12 = sustained virologic response 12 weeks after treatment completion.

### 3.6. Safety monitoring

Treatment safety was assessed through clinical documentation, laboratory data, and patient-reported symptoms obtained during routine visits at baseline, month 1, and EOT. AEs were defined as any new clinical symptom or laboratory abnormality emerging after treatment initiation. Serious AEs included hospitalization, life-threatening conditions, or treatment interruption attributable to therapy. Laboratory abnormalities were classified using the Common Terminology Criteria for Adverse Events (CTCAE v5.0).

### 3.7. Results of safety monitoring

No patients had their treatment discontinued due to treatment-limiting toxicity, and no serious AEs were recorded during treatment or follow-up. Mild and transient symptoms, such as fatigue, headache, or gastrointestinal complaints, reported in routine clinical notes, resolved without requiring further intervention. No clinically significant elevations in ALT or bilirubin were observed. Instead, a gradual improvement in markers of hepatocellular damage was observed throughout the treatment period. Platelet counts and INR values remained stable. No new cases of hepatic decompensation developed in patients with compensated cirrhosis.

### 3.8. Overall safety profile

Routine clinical monitoring data showed that both pangenotypic regimens were well tolerated. No treatment interruptions, dose modifications, or evidence of serious toxicity were observed.

## 4. Discussion

DAA therapies have become an integral part of the management of chronic hepatitis C, and pangenotypic regimens are recommended as first-line treatment options in current guidelines.^[1–3]^ In this study, a virological response was achieved in all patients who received treatment with sofosbuvir/velpatasvir/voxilaprevir or glecaprevir/pibrentasvir and for whom the SVR12 assessment was completed. No virological breakthrough or relapse was observed. This result is consistent with meta-analyses^[4,5]^ and real-world studies^[6–8]^ reporting high cure rates with pangenotypic regimens. However, given the limited number of patients and the absence of virological failure, this finding should be interpreted with caution.

In our study, we observed a decrease in ALT and total bilirubin levels and an increase in albumin levels. These findings suggest that hepatocellular damage may decrease and that liver synthetic function may improve during the course of treatment. Similar biochemical improvements following DAA therapy have also been reported in previous studies.^[9]^ Longitudinal elastography studies have demonstrated a reduction in liver stiffness following virologic cure, while studies evaluating fibrosis dynamics have indicated that structural improvement may continue over time following SVR.^[7,8,12]^ Although elastography or histological evaluation was not performed in this study, our biochemical findings are consistent with these data in the literature.

SVR12 was also achieved in certain patient groups previously considered difficult to treat. These groups included older patients, those who had previously received antiviral therapy, and patients with compensated cirrhosis. Previous antiviral exposure may influence treatment response, particularly in patients with prior treatment failure. In our cohort, only 5 patients (8.8%) were treatment-experienced, whereas the majority were treatment-naïve. All evaluable treatment-experienced patients achieved SVR12; however, the small number of previously treated patients precludes firm conclusions regarding the influence of prior antiviral exposure on treatment effectiveness. Real-world studies have reported that pangenotypic regimens demonstrate high efficacy across different viral subtypes, as well as in mixed or initially undetermined genotypes.^[10,11]^ Successful outcomes with DAA therapies have also been achieved in routine clinical practice in cohorts reported from Türkiye.^[12–14]^ Data from East Asia suggest that shorter treatment durations may be effective in selected patients with compensated cirrhosis.^[15]^ Multicenter studies comparing different pangenotypic regimens suggest that factors such as accessibility, tolerability, and ease of administration – rather than virologic efficacy – may be decisive in regimen selection.^[16]^ Studies showing improvements in patient-reported outcomes following DAA therapy also support the clinical benefits of successful treatment.^[17]^

In our study, the overall safety profile was favorable. No treatment discontinuations, dose adjustments, or serious AEs were observed; the mild symptoms reported resolved without requiring additional intervention. These tolerability findings are consistent with real-world and interventional studies evaluating pangenotypic regimens.^[4,6,16]^ Recent reviews emphasize the importance of improving access to screening and effective oral DAA therapy to achieve hepatitis C elimination goals.^[18]^ In more complex clinical settings, particularly among patients with prior DAA treatment failure, the sofosbuvir/velpatasvir/voxilaprevir regimen has been reported to yield favorable outcomes in terms of efficacy and safety.^[19]^ Economic modeling studies also support the cost-effectiveness of pangenotypic DAA regimens while maintaining high cure rates.^[20]^ Virological guidance identifies undetectable HCV RNA 12 weeks after treatment completion (SVR12) as the standard endpoint indicating virological cure.^[21]^

This study has several limitations. Its retrospective, single-center design and modest sample size may limit the generalizability of the findings. Because the primary effectiveness analysis was restricted to patients who completed treatment and had an available SVR12 assessment, the reported SVR12 rate represents a completer analysis and may overestimate effectiveness compared with an intention-to-treat approach. Because no virological failures occurred, subgroup analyses were descriptive, and comparative treatment effects could not be estimated. Long-term outcomes, such as hepatocellular carcinoma development and post-cure risk stratification, were not evaluated. Patient-reported quality of life was not assessed using a validated instrument, and serial elastography, histological evaluation, or other standardized measures of liver fibrosis were not available. Therefore, the observed biochemical improvements should not be interpreted as direct evidence of improved quality of life or fibrosis regression. These endpoints remain clinically important because the risk of hepatocellular carcinoma may persist after SVR in patients with advanced fibrosis.^[22]^ Studies from Türkiye have demonstrated sustained benefits of viral eradication in real-world cohorts,^[23]^ while broader analyses have reported clinical, economic, and quality-of-life benefits associated with achieving SVR.^[24]^ Despite these limitations, the inclusion of consecutive patients, complete follow-up within the analytic cohort, use of guideline-recommended pangenotypic regimens, and consistent findings across clinically relevant subgroups support the internal validity of the study.

In conclusion, in this single-center, real-world cohort, pangenotypic DAAs achieved SVR12 in all evaluable patients and demonstrated a favorable safety profile along with significant biochemical improvement during treatment. These findings support the use of simplified pangenotypic regimens in routine chronic HCV management while highlighting the need for larger multicenter studies with longer post-SVR follow-up.

## Author contributions

**Conceptualization:** Berfin Çirkin Doruk, Mustafa Uğuz.

**Formal analysis:** Mustafa Uğuz.

**Investigation:** Berfin Çirkin Doruk, Mustafa Uğuz.

**Methodology:** Mustafa Uğuz.

**Project administration:** Berfin Çirkin Doruk.

**Resources:** Berfin Çirkin Doruk.

**Writing – original draft:** Mustafa Uğuz.

**Writing – review & editing:** Berfin Çirkin Doruk, Mustafa Uğuz.
